# Two-Dimensional CdSe-PbSe Heterostructures and PbSe Nanoplatelets: Formation, Atomic Structure, and Optical Properties

**DOI:** 10.1021/acs.jpcc.1c09412

**Published:** 2022-01-17

**Authors:** Bastiaan B.V. Salzmann, Jur de Wit, Chen Li, Daniel Arenas-Esteban, Sara Bals, Andries Meijerink, Daniel Vanmaekelbergh

**Affiliations:** † Condensed Matter & Interfaces, Debye Institute for Nanomaterials Science, Utrecht University, 3508TA Utrecht, The Netherlands; ‡ EMAT and Nanolab Centre of Excellence, Antwerp University, 2020 Antwerp, Belgium

## Abstract

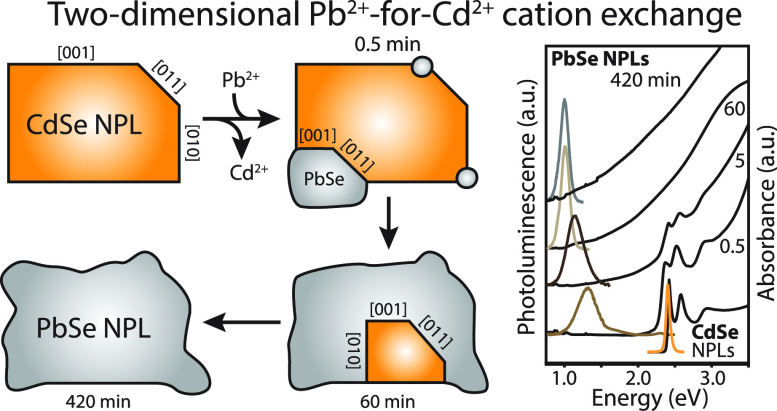

Cation exchange enables the preparation of nanocrystals (NCs), which are not reachable by direct synthesis methods. In this work, we applied Pb^2+^-for-Cd^2+^ cation exchange on CdSe nanoplatelets (NPLs) to prepare two-dimensional CdSe-PbSe heterostructures and PbSe NPLs. Lowering the reaction temperature slowed down the rate of cation exchange, making it possible to characterize the intermediary NCs ex situ with atomically resolved high-angle annular dark-field scanning transmission electron microscopy and optical spectroscopy. We observe that the Pb^2+^-for-Cd^2+^ cation exchange starts from the vertices of the NPLs and grows into the zinc blende CdSe (zb-CdSe) lattice as a rock salt PbSe phase (rs-PbSe), while the anion (selenium) sublattice is being preserved. In agreement with previous works on CdTe-PbTe films, the interfaces between zb-CdSe and rs-PbSe consist of shared {001} and {011} planes. The final PbSe NPLs are highly crystalline and contain protrusions at the edges, which are slightly rotated, indicating an atomic reconfiguration of material. The growth of PbSe domains into CdSe NPLs could also be monitored by the emission peak shift as a function of the exchange time. Temperature-dependent emission measurements confirm a size-dependent change of the band gap energy with temperature and reveal a strong influence of the anisotropic shape. Time-resolved photoluminescence measurements between 4 and 30 K show a dark-bright exciton-state splitting different from PbSe QDs with three-dimensional quantum confinement.

## Introduction

Cation exchange applied to colloidal nanocrystals transforms one compound into another, while the nanocrystal (NC) shape is often being preserved. Hence, this method enables the preparation of NCs of a certain compound and shape that cannot be reached by direct synthesis.^1−4^ Moreover, unconventional heterostructures can be obtained with epitaxial interfaces by a partial cation exchange, complementing the core/shell and core/crown systems obtained via a direct synthesis.^5^ Multiple works on the cation exchange of cadmium chalcogenide NCs have shown that the anion face-centered cubic (fcc) sublattice (Se^2–^, S^2–^) is being preserved^6−8^ and that the cation exchange in NCs occurs more swiftly than in their bulk counterparts, as the distance from a lattice position to the NC surface is only in the nanometer range.^9^

The synthesis procedures to perform a cation exchange on two-dimensional (2D) CdX (X = S, Se, Te) nanoplatelets (NPLs) have been classified as indirect or direct. Indirect methods are based on a two-step cation exchange via an intermediate copper chalcogenide phase. For example, PbS (ZnS) and PbSe/PbS (ZnSe/ZnS) core/shell NPLs have been prepared out of CdS and CdSe/CdS NPLs while retaining the original crystal shape.^10^ Alternatively, direct methods perform a cation exchange in a single step, although with less retention of shape. For instance, a Pb^2+^-for-Cd^2+^ cation exchange in a solution of PbBr_2_ and oleylamine has been demonstrated for different NC shapes such as quantum dots (QDs),^11,12^ nanowires,^13^ and NPLs of different thicknesses.^14,15^ Similarly, HgTe NPLs were prepared via a Hg^2+^-for-Cd^2+^ cation exchange via an HgCl_2_–OLAM (OLAM = oleylamine) procedure.^16^

Experimental procedures to perform a Pb^2+^-for-Cd^2+^ cation exchange on CdSe NPLs with PbBr_2_–OLAM have used 80 °C as the reaction temperature.^14,15^ Interestingly, this is a significantly lower temperature than that used for pseudospherical CdX (X = S, Se, Te) QDs of 2.5–6.5 nm in diameter; these systems required a higher reaction temperature between 80 and 190 °C to perform a full cation exchange.^12^ This is a strong indication that the activation energy needed to perform the Pb^2+^-for-Cd^2+^ cation exchange on 2D NPLs is lower than that of QDs. In the case of NPLs, it is also of large interest to see in which way cation exchange proceeds; the route via the top and bottom surface would minimize the travel distance in the lattice, but it is known that the top and bottom surfaces are well-passivated by Cd-oleate.^17^ The route via the vertical side facets is much longer on average, but these facets are much less stable than the top and bottom surfaces. In this respect, we remark that a thermochemical reconfiguration of CdSe NPLs into quantum rings also occurs via the vertical facets and vertices.^18^

By slowing the rate of cation exchange we could extract and characterize intermediate samples and, thus, NPLs in which both CdSe and PbSe are present. Earlier works reported core–shell and hemisphere Janus PbSe/CdSe heteroNCs with epitaxial interfaces^12^ and CdS tetrapods on which PbSe tips are formed.^19^ Neither the shape, atomic structure, and optical properties of 2D CdSe-PbSe heteroNPLs nor the microscopic details of the cation exchange have been studied.^14^

In addition, the optoelectronic properties of PbSe/CdSe heteronanocrystals are of high interest. Exploitation of different band alignments in core/shell PbSe/CdSe and multishell PbSe/CdSe/CdS QDs successfully showed upconversion from near-infrared (NIR) to visible light.^20,21^ Recent work on type-I PbSe/CdSe dot-on-plate heterostructures established rapid transfer of excitations from CdSe NPLs to the attached PbSe QDs, thereby effectively using the high absorption cross-section of the CdSe NPLs.^22^

Here, we investigate the conversion of 4.5 monolayer (ML) thick CdSe NPLs into CdSe-PbSe heterostructures and PbSe NPLs via a direct Pb^2+^-for-Cd^2+^ cation exchange with PbBr_2_ and oleylamine. We lowered the reaction temperature from the reported 80 °C to 40 and slowed the reaction so much that we could extract aliquots with 2D PbSe-CdSe heterostructures. The atomic structure of these intermediate reaction products reveals the crystallography of cation exchange in these 2D NPLs. Moreover, the 2D CdSe-PbSe heterostructures have interesting optical properties with swift exciton energy transfer from the CdSe lattice to the PbSe domains.

## Experimental section

### Chemicals

1-Butanol (BuOH, anhydrous, 99.8%), cadmium acetate (Cd(OAc)_2_, 99.995%), cadmium acetate dihydrate (Cd(OAc)_2_·2H_2_O, ≥98.0%), cadmium nitrate tetrahydrate (Cd(NO_3_)_2_·4H_2_O, 98%), methanol (MeOH, anhydrous, 99.8%), 1-octadecene (ODE, technical grade 90%), oleic acid (OA, technical grade 90%), oleylamine (OLAM, technical grade 70%), sodium myristate (≥99%), and tetrachloroethylene (TCE, anhydrous, ≥99%) were bought from Sigma-Aldrich. *n*-Hexane (anhydrous), selenium (200 mesh, 99.99%), and tri-*n*-butyl-phosphine (TBP, 95%) were bought from Alfa Aesar, STREM Chemicals, and Acros Organics, respectively. ODE, OLAM, and OA were degassed in a Schlenk line before use.

### Synthesis of 4.5 ML CdSe NPLs

CdSe NPLs with a thickness of 4.5 MLs were prepared in a N_2_-filled glovebox via an earlier reported synthesis method of Bertrand et al.^23^ To obtain NPLs with a square aspect ratio, a mixture of 50/50 mol % Cd(OAc)_2_·2H_2_O/Cd(OAc)_2_ powder was used. Afterward, the mixture was washed with a 1:2 mixture of MeOH/BuOH. The 4.5 ML NPLs were subsequently separated from QDs and 3.5 ML thick CdSe NPLs via a size-selective precipitation by the addition of small amounts of MeOH/BuOH and centrifugation. The desired 4.5 ML NPLs were finally redispersed in hexane, and multiple batches were combined to yield a concentrated stock dispersion with an orange color.

### Conversion of 4.5 ML Thick CdSe NPLs into CdSe Quantum Rings

CdSe NPLs were converted into CdSe quantum rings by a previously reported procedure.^18^ Briefly, selenium was dispersed in OLAM to yield a concentration of 7.9 mg Se/mL OLAM. 1.0 mL of CdSe NPLs with an absorbance of 0.2 (in a 1 cm cuvette) at the first exciton transition after being diluted 300 times was precipitated and redispersed in 3 mL of ODE and 1.5 mL of OLAM. The redispersed NPLs were heated to 80 °C for 10 min to allow the remaining hexane to evaporate. Thereafter, 200 μL of the Se-OLAM mixture was added and heated to 155 °C for 10 min in an 8 mL reaction vial. Next, 200 μL of TBP was added while the mixture was quickly heated to 220 °C. After the solution was allowed to cool, the mixture was washed once with a 1:2 solution of MeOH/BuOH and redispersed in hexane.

### Pb^2+^-for-Cd^2+^ Cation Exchange

The Pb^2+^-for-Cd^2+^ cation exchange on the 4.5 ML CdSe NPLs was performed using a previously reported protocol.^14^ First, a PbBr_2_–OLAM mixture was prepared in a N_2_-filled glovebox by mixing 3 mL of ODE with 1 mL of OLAM, together with 24 mg of PbBr_2_ (0.065 mmol) powder. This mixture was heated under vigorous stirring to 100 °C for 15 min, yielding a colorless solution. Second, the PbBr_2_–OLAM mixture was cooled to the desired reaction temperature, that is, 80, 60, 40, or 25 °C. Then, 1.0 mL of the 4.5 ML CdSe NPLs were quickly added from the stock solution, which had an absorbance of 0.2 (in a 1 cm cuvette) at the first exciton transition after being diluted 300 times. Directly after the addition of the CdSe NPLs, the color of the mixture turned from orange to brown and finally into black. Lowering the reaction temperature slowed the rate of the Pb^2+^-for-Cd^2+^ cation exchange, visible as a more gradual color change. Aliquots of ∼300 μL were taken with Pasteur pipettes during the exchange reaction and were immediately quenched in a mixture of 400 μL of TCE and 100 μL of OA to prevent agglomeration. It was found that this volume was enough to perform washing and further characterization of the intermediate heterostructured NCs. The final product dispersion was washed by the addition of 200 μL of OA and 4 mL of MeOH/BuOH, centrifuged and redispersed in TCE.

### Optical and Structural Characterization

Photoluminescence measurements were performed on an Edinburgh Instruments FLS920 spectrometer equipped with a TMS300 monochromator, 450 W Xe lamp, thermoelectrically cooled Hamamatsu R928 PMT detector, and a liquid N_2_ cooled R5509-72 NIR PMT for wavelengths beyond 825 nm. The recorded emission spectra were corrected for the spectral responsivity of the detectors and monochromators. Photoluminescence decay curves were recorded with a pulsed Coherent 45 mW OBIS LX 445 nm laser (modulated with an Agilent function generator) and an R5509-72 NIR PMT. Cryogenic measurements were performed in a continuous-flow liquid helium cryostat from Oxford Instruments. UV/Vis absorption spectra were measured on a PerkinElmer 950 UV/vis/NIR spectrophotometer. Transmission electron microscopy (TEM) samples were made by drop-casting a diluted dispersion of NCs on carbon-coated TEM copper grids. Bright-field (BF-TEM) and high-angle annular dark-field scanning transmission electron microscopy (HAADF-STEM) images were taken on a Talos F200X from FEI operating at 200 keV. High-resolution HAADF-STEM imaging was performed on an aberration-corrected Titan electron microscope from Thermofisher operating at 300 keV. To minimize structural changes of NCs during imaging, a low beam current of ∼5 pA was used with relatively low magnifications.

## Results

Heterostructured CdSe-PbSe NPLs and PbSe NPLs are prepared by performing Pb^2+^-for-Cd^2+^ cation exchange with PbBr_2_ and oleylamine (OLAM) on 4.5 ML thick CdSe NPLs following an earlier reported procedure of Galle et al.^14^ On the basis of the lattice enthalpies of CdSe and PbSe (Δ*H*_latt_ of, respectively, 3310 and 3144 kJ/mol), one would not expect to observe a Pb^2+^-for-Cd^2+^ cation exchange because of the slightly lower lattice energy (and thus lower stability) of the final PbSe lattice.^3,24^ However, the driving force can be explained by a combination of an excess of Pb^2+^ cations and the favorable solvation energy of Cd^2+^ with OLAM. We estimated a Pb^2+^/Cd^2+^ ratio of 7.8:1 during the exchange reaction (see Section S1), explaining that the equilibrium lies toward the incorporation of Pb^2+^ cations into the selenium sublattice. The strong solvation energy of Cd^2+^ can be understood in terms of the hard–soft acid–base (HSAB) theory, which predicts the affinity among ions and solvents. In here, a complex of a hard acid with a hard base (i.e., Cd-OLAM) is more stable than a soft acid with a hard base (i.e., Pb-OLAM), meaning that Cd-OLAM is energetically more favorable than Pb-OLAM. Under the circumstances mentioned above, the Pb^2+^-for-Cd^2+^ exchange is therefore favored in these CdSe NPLs.^3,13^

**Figure 1:**
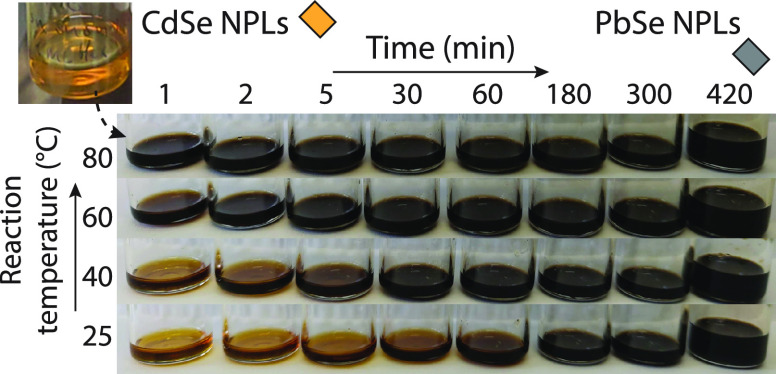
Investigating the influence of the reaction temperature on the Pb^2+^-for-Cd^2+^ cation exchange of CdSe NPLs with PbBr_2_–OLAM. Photographs of sample vials containing the aliquot dispersions taken at specific intervals using different reaction temperatures (80, 60, 40, and 25 °C). The reaction temperature of 40 °C was chosen for further investigation.

Figure 1 shows photographs of samples taken during the conversion at 80, 60, 40, and 25 °C for up to 420 min of the reaction. As the exchange reaction proceeds very fast at 80 °C, the dispersion turns black after 1 min of reaction, indicating a complete Pb^2+^-for-Cd^2+^ cation exchange. When the reaction temperature is lowered from 80 to 40 and 25 °C, less dramatic color changes to yellow-brown dispersions are observed during the first stages of reaction; this suggests that intermediate CdSe-PbSe heterostructures can be isolated. Regardless of the reaction temperature, all dispersions have turned completely black after 180 min, pointing to a complete Pb^2+^-for-Cd^2+^ exchange of the NCs. We chose to study the aliquot series prepared at 40 °C further with HAADF-STEM and optical spectroscopy.

### Ex Situ Monitoring of Cation Exchange with Electron Microscopy and Optical Spectroscopy

**Figure 2:**
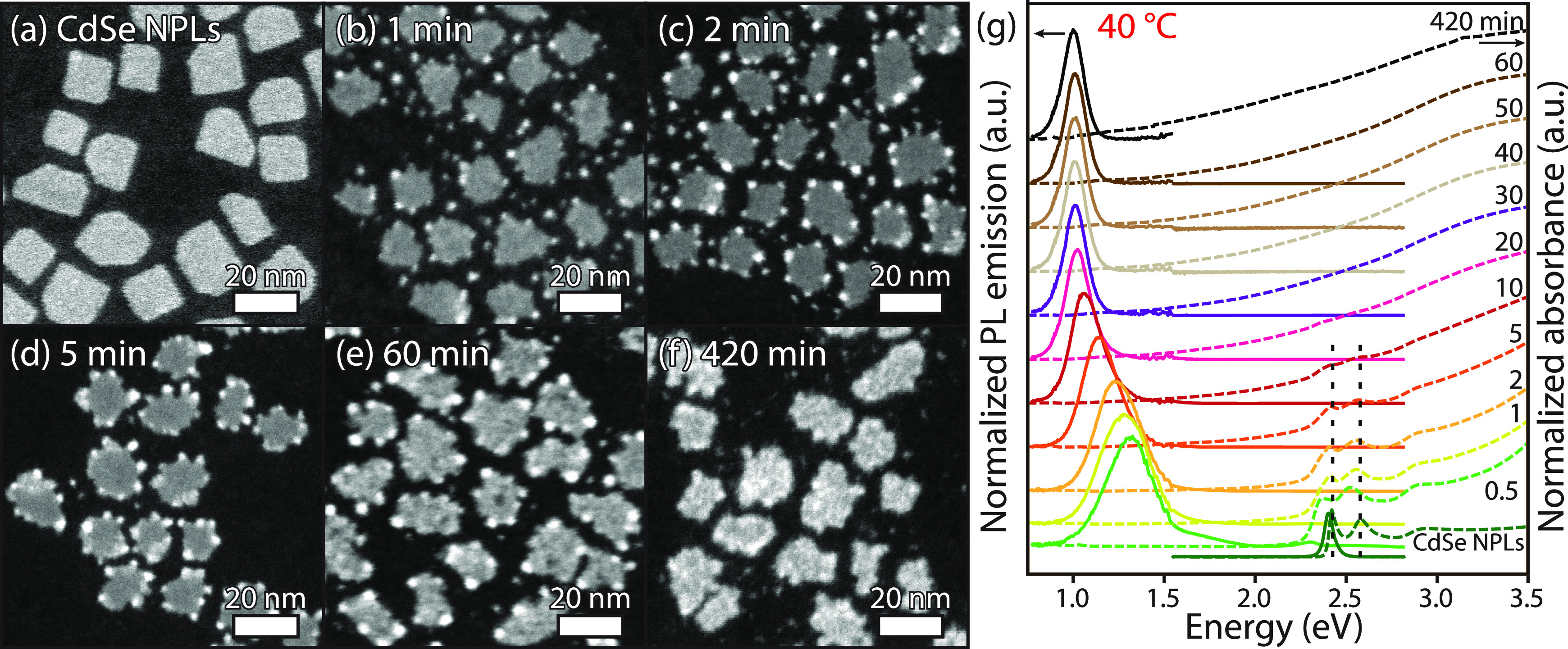
Structural and optical characterization of intermediate CdSe-PbSe heterostructures and PbSe NPLs prepared by a Pb^2+^-for-Cd^2+^ cation exchange on CdSe NPLs. (a–f) HAADF-STEM images of CdSe NPLs (a) and aliquots taken after cation exchange for 1, 2, 5, 60, and 420 min of reaction (b–f). All scale bars are 20 nm. (g) Corresponding absorption (dashed lines) and emission (continuous lines) spectra of the intermediate structures and final PbSe NPLs. The dashed vertical lines indicate the (HH,e) and (LH,e) absorption features of 4.5 ML CdSe NPLs. Photoluminescence emission was recorded upon excitation with 400 or 405 nm (3.10 or 3.06 eV); the absorption and emission spectra were normalized afterward. The discontinuity in the emission spectra at 1.55 eV (800 nm) is caused by a change of detector to the NIR-sensitive PMT.

Figure 2 shows the HAADF-STEM images and corresponding absorption (dashed lines) and emission (continuous lines) spectra of the CdSe-PbSe heterostructures prepared at 40 °C. The time evolution of absorption and emission spectra from other reaction temperatures (80, 60, and 25 °C) can be found in Figure S2. The advantage of using HAADF-STEM for this type of NCs is that an element-specific contrast is obtained, as the intensity scales with the atomic number *Z* (also called *Z-imaging*).^25^ Lead-rich regions will appear with a higher contrast (i.e., brighter) than cadmium-rich domains, which will have a lower contrast (i.e., darker), different from bright-field TEM (Figure S3).

As can be observed in Figure 2a, the CdSe NPLs appear with a homogeneous contrast, indicating uniform thickness within the NCs.^26^ The absorption spectrum (Figure 2g) shows the characteristic features of the heavy hole–electron (HH,e) and light hole–electron (LH,e) transitions at 2.42 and 2.58 eV, together with a narrow emission band slightly red-shifted from the (HH,e) transition, both in line with previous reports on 4.5 ML thick CdSe NPLs.^23,26^

In addition, bright dots are present separate from the CdSe NCs and at the edges of the NCs in the HAADF-STEM image of the aliquot taken after 1 min (Figure 2b), which we attribute to nucleation of separate PbSe NCs and small PbSe domains in the CdSe NPLs.These bright dots are absent in the center of the NCs, proving that the cation exchange starts at the vertical facets from which the Pb^2+^ cations migrate into the CdSe crystal lattice. Moreover, this also implies that the cadmium-terminated top and bottom facets of the CdSe NPLs are fully protected by ligands and that a lower activation energy is required for the Pb^2+^-for-Cd^2+^ exchange at the vertices. This finding is similar to the observation of pseudospherical PbSe quantum dots^22^ and metallic nanoparticles^27^ grown on the edge corners of CdSe NPLs, showing that these locations are also preferred sites for growth of other compounds.

The (HH,e) and (LH,e) transitions of CdSe are also clearly visible in the absorption spectrum of the 0.5 min aliquot, although slightly shifted to lower energies in comparison to pure 4.5 ML CdSe NPLs. The redshift can be explained by the replacement of native oleate and acetate ligands to bromide ions. Previous research has shown that the native ligands apply strain in the thickness direction, yielding a tetragonal distortion of the crystal lattice.^28,29^ The replacement with halides (i.e., Br^–^, Cl^–^, and I^–^) partially releases the strain, resulting in a slight increase in thickness and thus a relaxation of quantum confinement. Although features from the PbSe domains are not visible in the linear absorption spectrum, plotting the data on a logarithmic scale (Figure S2) shows enhanced absorption between 2.00 and 2.25 eV, indicating the presence of PbSe domains. The emission spectrum displays a strongly red-shifted and broad photoluminescence peak at 1.33 eV, which is assigned to emission from PbSe domains. Moreover, a weak emission peak is visible at the low-energy side of the (HH,e) transition, possibly from a minor population of unchanged CdSe NPLs.

HAADF-STEM images of samples taken after 2 and 5 min (Figure 2c,d) reveal a slightly higher number of Pb-rich domains, while the homogeneous contrast in the centers remains unaffected. Next to the (HH,e) and (LH,e) features from CdSe in the absorption spectrum, an enhanced absorption between 1.25 and 2.25 eV is present along with a redshift of the emission band to 1.29 eV, indicating further growth of the PbSe domains. The absence of photoluminescence from CdSe in the heterostructures can be explained by the fast electron–hole excitation energy to PbSe, in agreement with previous results for PbSe-CdSe dot-on-plate heterostructures in which a rapid energy transfer from CdSe to PbSe was observed on a picosecond time scale.^22^

The sample taken after 60 min reveals NCs with inhomogeneous contrast in the centers and bright dots at the edges. Energy-dispersive X-ray (EDX) spectroscopy in the BF-TEM mode (Figure S4) shows that these structures have been fully converted into PbSe, indicating that the bright dots are PbSe regions, which are thicker. A longer reaction time (420 min of reaction) results in a further shift of absorption and emission to lower energies. Moreover, selected area electron diffraction (SAED) measurements confirm the presence of the rock salt crystal structure of the PbSe NPLs (Figure S5), in accordance with X-ray diffraction (XRD) measurements of Galle et al.^14^

To summarize, it is evident from the optical and structural characterization that cation exchange starts from the vertices of the CdSe NPLs, instead of the top and bottom facets. As time progresses, the Pb^2+^-for-Cd^2+^ cation exchange causes a continuing lateral growth of the PbSe domains into the inner part of the NPL, until full PbSe NPLs are obtained. Further investigation of the crystallinity and interfaces of the CdSe-PbSe heterostructures prepared at 40 °C with high-resolution HAADF-STEM will be presented below.

### Detailed Investigation of the CdSe-PbSe Interface with High-Resolution HAADF-STEM

Colloidal CdSe-PbSe NCs, such as pseudospherical quantum dots and nanorods,^30,31^ show (001) and (111) heterointerfaces between the zinc blende (zb) and rock salt (rs) crystal domains. Moreover, cation exchange in these systems proceeds along a vacancy-assisted pathway, in which the cations are exchanged layer-by-layer while the anion sublattice is preserved.^6,7,31,32^ Similarly, zb-rs interfaces with continuous anion lattices have also been observed in films prepared by solid-state techniques, including CdTe-PbTe,^33−35^ PbSe-InAs,^36^ and InGa_1–*x*_As_*x*_-ErAs.^37^ As their preparation requires elevated growth temperatures over 200 °C, the interfaces are almost defect-free and consist of epitaxially connected (001) and (011) lattice planes.

**Figure 3:**
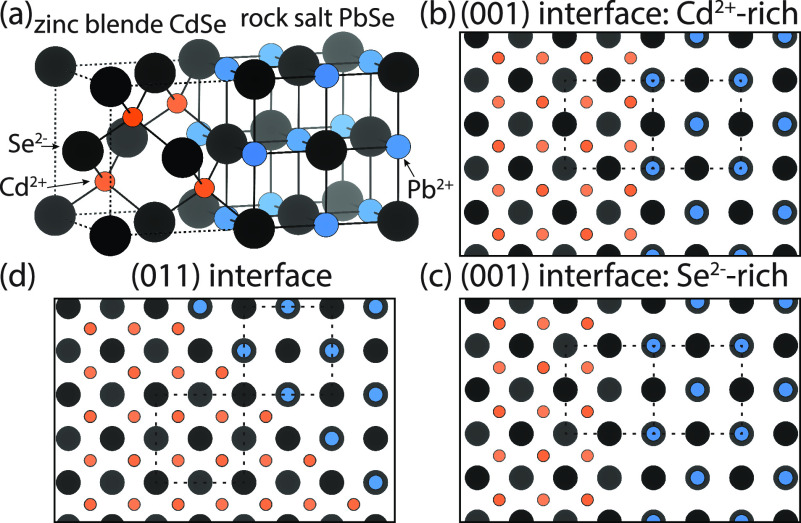
Schematic depictions of heterostructures from zb-CdSe and rs-PbSe with a continuous (anion) selenium sublattice (black spheres). (a) Connected unit cells of zb-CdSe and rs-PbSe showing the continuous selenium sublattice with the Cd^2+^ (orange) and Pb^2+^ ions (blue) located in the tetrahedral and octahedral holes, respectively. (b, c) Polar (001) CdSe-PbSe interfaces seen along the [100] top view direction, which are rich in either cadmium (b) or selenium (c), depending on the termination of the zb-CdSe plane. Dashed squares indicate the unit cells of zb-CdSe and rs-PbSe. (d) Nonpolar (011) interface between zb-CdSe and rs-PbSe seen along the [100] top view direction.

Figure 3a depicts connected unit cells of zinc blende CdSe (zb-CdSe) and rock salt PbSe (rs-PbSe) with a continuous selenium sublattice and a shared (001) interface. As can be seen, in the case of zb-CdSe, cadmium and selenium are tetrahedrally coordinated, whereas for rs-PbSe both lead and selenium have an octahedral coordination. Because of the small lattice mismatch between the two crystal lattices (<1%, lattice constants of 6.077 and 6.128 Å for zb-CdSe and rs-PbSe), epitaxial connections between the two crystal lattices are possible.^32,38^ We sketched the possible zb-CdSe–rs-PbSe interfaces seen along the [100] top-view direction in Figure 3b–d; this should help the HAADF-STEM analysis of the 2D PbSe-CdSe NPLs presented below. In the case of shared (001) interfaces, the termination of zb-CdSe determines if the interface is either cadmium- or selenium-rich (Figure 3b,c). Next to (001) interfaces, charge neutral nonpolar (011) interfaces have been observed (Figure 3d).

**Figure 4:**
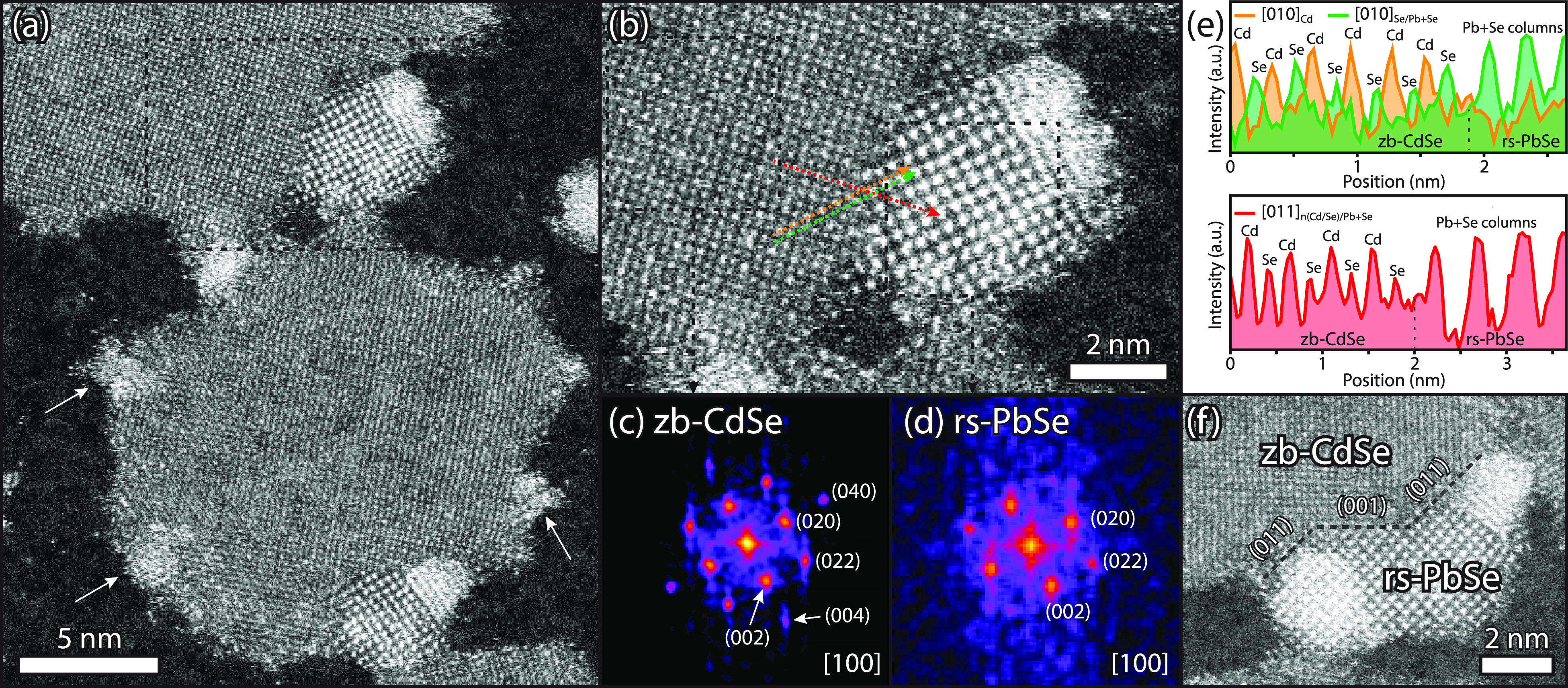
Analysis of high-resolution HAADF-STEM images of CdSe-PbSe NPLs after 0.5 min of reaction time at 40 °C. (a) HAADF-STEM image, showing monocrystalline CdSe NPLs with PbSe domains at the edges with a high contrast. (b) Enlarged view of (a) showing the atomic ordering of both zb-CdSe and rs-PbSe with a (011) interface between the two crystal domains. (c, d) FFT patterns of two selected regions in (b), indicating the high crystallinity of zb-CdSe and rs-PbSe, respectively. (e) Intensity profiles along the colored lines in (b), confirming both the crystallinity and presence of a (011) interface between zb-CdSe and rs-PbSe. (f) HAADF-STEM image of a larger PbSe domain showing the presence of both (001) and (011) interfaces.

Atomically resolved HAADF-STEM was used to investigate the crystallinity and interfaces in the (partially) exchanged NCs. As we observed the structural reconfiguration of a material due to beam damage after imaging (Figure S6), relatively low beam currents and magnifications were used to minimize these effects. Figure 4 shows the results of CdSe-PbSe heterostructures after a cation exchange for 0.5 min. Similar to the low-resolution HAADF-STEM images (Figure 2), the CdSe and PbSe domains appear, respectively, with a low and high contrast but now showing the atomic ordering in most regions. As can be observed in Figure 4a, several small PbSe regions with a high contrast are visible in which the cation exchange process just started (indicated with white arrows), together with larger PbSe domains showing visible crystallinity. We chose to analyze the CdSe-PbSe interface at the top part of the image; an enlarged view this region is shown in Figure 4b. To quantify the degree of crystallinity, fast Fourier transform (FFT) patterns were calculated; see Figure 4c,d. The FFT pattern of the CdSe region shows the characteristic reflections from zb-CdSe up to the (040) lattice planes with [100] in zone axis, in agreement with literature.^26^ The FFT pattern of the high-contrast region shows the characteristic reflections of rs-PbSe with [100] in a zone axis, in agreement with the previous report of Galle et al.^14^ The (002) and (020) reflections from both crystal structures show similar directions, and this forms a first indication that the selenium sublattice is preserved during the Pb^2+^-for-Cd^2+^ cation exchange.

Intensity profiles were taken along the [010] and [011] directions (colored arrows in Figure 4b) to investigate the interface and epitaxial connection of the two crystal domains. To reveal the difference between the cadmium and selenium columns along the [010] direction, two profiles were taken with the spacing of a half unit cell, indicated as [010]_Cd_ and [010]_Se/Pb+Se_ in Figure 4e. As can be seen, the [010]_Cd_ profile (orange line) exhibits a significantly higher scattering intensity than the [010]_Se/Pb+Se_ profile (green line) in the zb-CdSe region, and the low-intensity peaks are located between the high-intensity peaks. From this, it is evident that these directions correspond to cadmium and selenium columns in the zb-CdSe crystal lattice. When following both intensity profiles into the rs-PbSe region, clear peaks of columns containing both Pb and Se are visible in [010]_Se/Pb+Se_, while these are absent in the [010]_Cd_ profile. This observation demonstrates that the selenium lattice continues along the [010]_Se/Pb+Se_ direction and thus that the selenium lattice is preserved during the cation exchange. Additional proof for the preservation of the selenium lattice is unveiled by the [011]_n(Cd/Se)/Pb+Se_ intensity profile (red line). Here, peaks with an alternating intensity are visible in the zb-CdSe domain, which shows the presence of alternating Cd and Se columns. High-intensity peaks are visible upon following the profile into rs-PbSe, corresponding to columns containing both Pb and Se.

Next to the presence of (011) interfaces, we observed several zb-CdSe-rs-PbSe NCs with (001) interfaces (see Figure 3d). Figure 4f shows an HAADF-STEM image with a slightly larger domain of rs-PbSe containing both (001)- and (011)-type interfaces. From the above observations, we can conclude that the selenium sublattice is being preserved during the Pb^2+^-for-Cd^2+^ exchange, in agreement with previous works on cation exchange. We also remark here that our results suggest that the interfaces are very clear-cut and both crystal phases are pure. For instance, there is no indication that the CdSe phase contains Pb^2+^ “front runners” away from the interfacial region.

**Figure 5:**
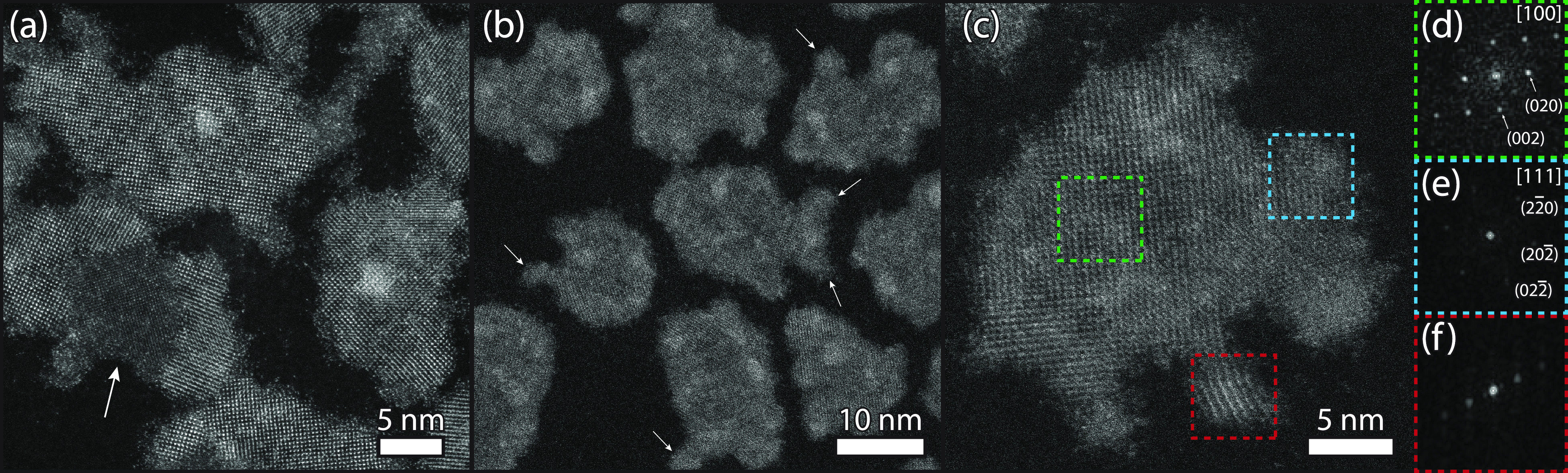
High-resolution HAADF-STEM images of Pb^2+^-for-Cd^2+^ cation-exchanged CdSe NPLs obtained after 60 and 420 min of reaction at 40 °C. (a) CdSe-PbSe heterostructures after a cation exchange for 60 min show an almost full Pb^2+^-for-Cd^2+^ cation exchange. The region with a lower contrast (indicated with an arrow) indicates the presence of zb-CdSe, showing an incomplete cation exchange. (b) PbSe NPLs after a cation exchange for 420 min, containing protrusions at the edges of the NPLs (indicated with arrows). (c) Enlarged view of a single PbSe NPL with multiple protrusions, together with corresponding FFT patterns of different regions (colored boxes) in (d–f). (d) FFT pattern of the center of the PbSe NPL, showing rs-PbSe along the [100] zone axis. (e) FFT pattern of a protrusion with the characteristic {022} reflections of rs-PbSe but now with the [111] zone axis. (f) FFT pattern of a protrusion highly tilted where only the {111} reflection is visible.

High-resolution HAADF-STEM images of CdSe-PbSe heterostructures after a cation exchange for 60 and 420 min are shown in Figure 5. Most of the particles have fully been exchanged after 60 min of reaction (Figure 5a), as columns of rs-PbSe are visible with a high crystallinity. Nevertheless, several NCs show incomplete conversion, as lower contrast domains of zb-CdSe are present (indicated with an arrow). Differently, cation exchange has been completed after 420 min of reaction, resulting in NCs with a similar shape without any low-contrast zb-CdSe domains (Figure 5b). The absence of zb-CdSe (<1% give the detection limit) was confirmed with EDX in the HAADF-STEM mode, showing that no cadmium was left in the NCs (Figure S7).

Interestingly, both the 60 and 420 min samples show protrusions at the edges of the NCs, indicated in the 420 min sample with white arrows (Figure 5b). Although we have shown that the selenium sublattice is being preserved during the cation exchange process, this observation suggests that an atomic reconfiguration takes place to a more energetically stable rock salt crystal shape at the edges of the NCs. We found that these smaller domains are rotated relative to the NPL, see the FFT patterns of different regions of a single PbSe NPL in Figure 5c–f. The FFT pattern from the center of the NPL shows the typical reflections of rs-PbSe (Figure 5d). In contrast, the FFT pattern of the protrusion in the dashed blue box exhibits a different orientation, as the [111] direction in the zone axis and contains the corresponding {022} reflections (Figure 5e). The FFT pattern of the red box only shows the presence of the {111} reflections indicating that the crystal structure of the rs-PbSe protrusion has been rotated by certain α and β angles. Additional BF-TEM images show that the thickness of the NPLs is mostly preserved, resulting in PbSe NPLs composed of six to nine layers of rs-PbSe (Figure S8).

### Applicability of Pb^2+^-for-Cd^2+^ Cation Exchange on Other Crystal Shapes: The Case of CdSe Quantum Rings

**Figure 6:**
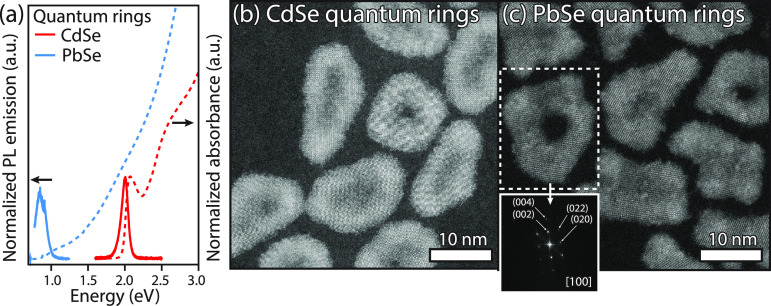
Characterization of PbSe quantum rings obtained by a Pb^2+^-for-Cd^2+^ exchange of CdSe quantum rings. (a) Absorption (dashed lines) and emission (solid lines) spectra of CdSe and PbSe quantum rings. (b) High-resolution HAADF-STEM image of zb-CdSe quantum rings. (c) High-resolution images of rs-PbSe quantum rings after a cation exchange. (inset) FFT pattern of a single PbSe quantum ring, confirming the rs-PbSe crystal lattice.

To show that a Pb^2+^-for-Cd^2+^ cation exchange with PbBr_2_–OLAM is applicable to other shapes of CdSe NCs, the exchange procedure was performed on zb-CdSe nanorings with a toroidal shape.^18,39^Figure 6a shows the obtained optical absorption and emission spectra, together with high-resolution HAADF-STEM images of the corresponding CdSe and PbSe quantum rings. In agreement with previous reports, the photoluminescence of the CdSe quantum rings is centered around 2.0 eV, while the first absorption peak is slightly blueshifted to 2.05 eV (Figure 6a). After the Pb^2+^-for-Cd^2+^ exchange at 80 °C for 420 min, both the absorption and emission band bands have been shifted to lower energies, similar to the Pb^2+^-for-Cd^2+^ cation exchange on CdSe NPLs discussed before. Apart from a shoulder close to the absorption onset at 1.0 eV, the absorption spectrum is featureless. The photoluminescence emission peak is redshifted from the absorption onset and is centered at 0.87 eV.

Figure 6b shows a representative HAADF-STEM image of CdSe quantum rings. As can be seen, the NCs have a toroidal shape and are highly crystalline, in agreement with our previous work.^18^ Although several particles are fully perforated, others have a membrane in the center with a thickness of the original CdSe NPL.^18^ After the cation exchange, the shape of the NCs is mostly preserved (Figure 6c). The FFT pattern of a single PbSe quantum ring (inset) shows that the NCs are highly crystalline, as the characteristic reflections from rs-PbSe are observed. EDX in the HAADF-STEM mode confirms these results, as a minimal amount of cadmium is observed (Figure S9). To summarize, we can conclude that a Pb^2+^-for-Cd^2+^ cation exchange is also applicable to other shapes of NCs, while the shape of the original particle is being preserved.

### Investigating the Optical Properties of Two-Dimensional CdSe-PbSe Heterostructures and PbSe NPLs at Cryogenic Temperatures

Important insights in the cation exchange and growth of PbSe domains into the CdSe NPLs can also be obtained from optical spectroscopy, as quantum confinement strongly influences the optical properties of PbSe nanostructures. Therefore, we selected three samples (0.5, 5, and 420 min of a Pb^2+^-for-Cd^2+^ cation exchange) with different PbSe lateral domain sizes and investigated these with a combination of time-resolved and temperature-dependent optical spectroscopy.

**Figure 7:**
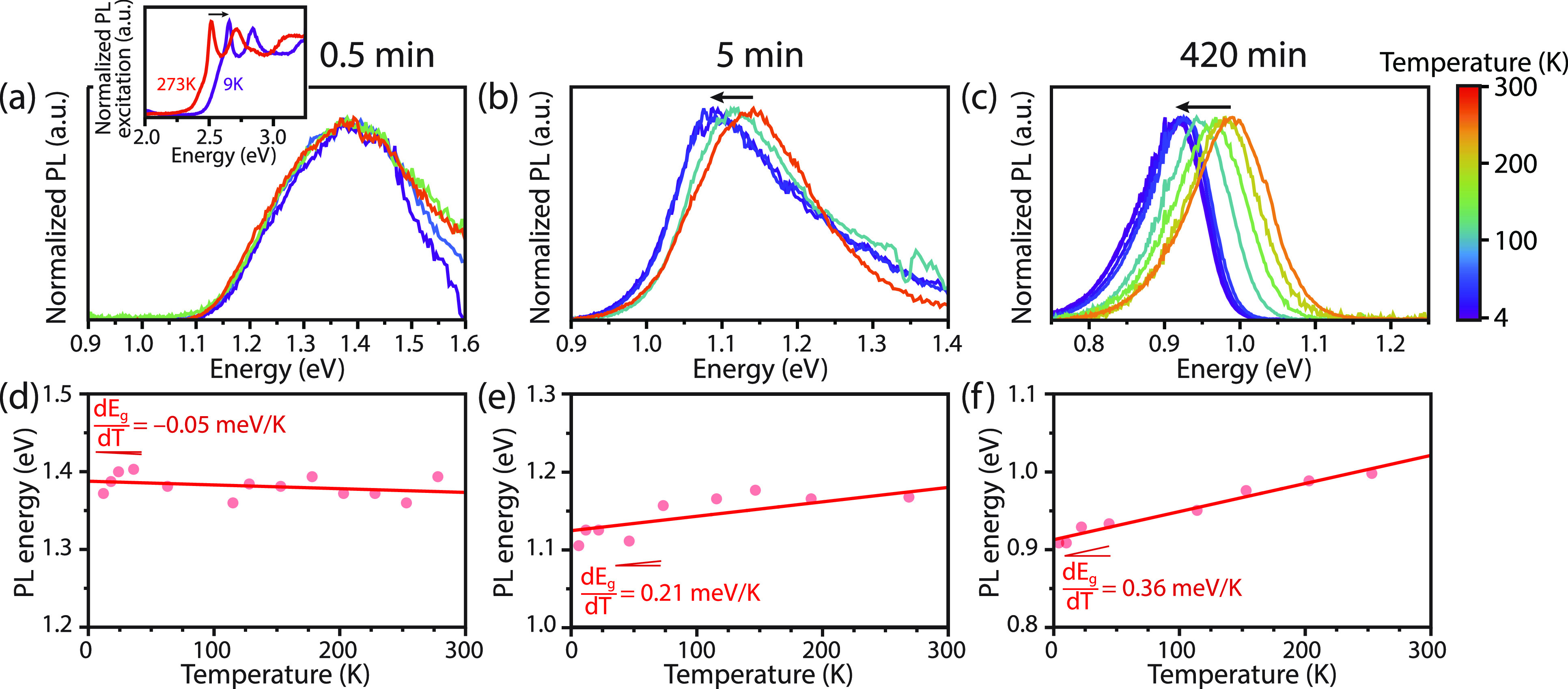
Photoluminescence emission spectra of CdSe-PbSe NCs after (a) 0.5, (b) 5, and (c) 420 min of Pb^2+^-for-Cd^2+^ cation exchange at 40 °C, recorded at temperatures between 300 and 4 K. (inset) Excitation spectra recorded at 9 and 273 K with an emission energy of 1.85 eV. (d–f) Corresponding peak position versus the temperature. For the smallest PbSe domain size/shortest cation exchange time (0.5 min), there is a negative change in *E*_g_, while for the longer periods (5 and 420 min) there is an increase in the band gap with increasing temperature. The red line represents a linear fit, from which the temperature shift of the exciton energy  is determined.

Temperature-dependent excitation and emission spectra were recorded for CdSe NPLs after 0.5 min of Pb^2+^-for-Cd^2+^ cation exchange at 40 °C, showing a broad emission band around 1.4 eV at room temperature (Figures 6a and S10). High-resolution HAADF-STEM imaging (Figure 4) revealed that this sample contained an average rs-PbSe domain size of 4.4 ± 3.1 nm^2^, together with PbSe QDs with a diameter of ∼2 nm. The observed peak position is similar to the exciton energy reported for 2 nm diameter PbSe QDs and is much higher than the 0.27 eV bulk band gap value of PbSe, indicating a three-dimensional (3D) quantum confinement of the incorporated PbSe domains.^40^ The large width of the emission band is explained by inhomogeneous broadening caused by the relatively large size inhomogeneity of the rs-PbSe domains. Evidence for strong coupling of the PbSe domains to the CdSe NPLs is clearly visible in the excitation spectra of the PbSe emission (see inset of Figure 7a and Figure S10), which reveals the strong and characteristic absorption features of 4.5 ML CdSe NPLs next to a weak onset below 2.45 eV typical for above bandgap PbSe absorption. The weak absorption below 2.45 eV probably involves both a direct excitation of PbSe domains and separate PbSe NCs. An efficient energy transfer from CdSe NPLs to PbSe domains almost completely quenches the CdSe NPL exciton emission already after 0.5 min of cation exchange, similar to the previously reported PbSe-dot-on-CdSe-NPL heterostructures.^22^ When cooled to 4 K the excitation lines of CdSe NPLs shift to higher energies due to the lattice contraction of the zb-CdSe crystal lattice, as has been shown for pure 4.5 ML CdSe NPLs.^41^

The PbSe domains after 5 min of cation exchange at 40 °C have grown to 24.9 ± 22.4 nm^2^, as determined from high-resolution HAADF-STEM images. Given the large exciton-Bohr radius of PbSe (*a*_0_ = 46 nm), the larger lateral size reduces the quantum confinement along the lateral directions, thereby redshifting the emission peak to ∼1.15 eV (Figure 7b). The excitonic feature around 1.1 eV in the absorption spectra is relatively broad and indicates that thickness inhomogeneity (Figure S8) and variations in quantum confinement in a lateral direction causes an inhomogeneous broadening. Upon comparing this result to literature values of PbSe QDs with a diameter of 5 nm, which show emission at 0.7–0.8 eV,^40,42^ the higher emission energy of these CdSe-PbSe heterostructures can be explained by a strong confinement in the thickness direction, in line with our finding that the crystal thickness is being preserved during cation exchange (see Figure S8).

After 420 min of reaction, the Pb^2+^-for-Cd^2+^ cation exchange is complete (see above), and PbSe NPLs with lateral sizes over 10 nm have been formed. As a result, the further decrease of quantum confinement along the lateral directions causes the emission peak to shift to ∼1.0 eV (Figure 7c), a similar energy to what has been reported before by Galle et al.^14^ In spite of the presence of protrusions in these PbSe NPLs (Figure 5c), the emission band is relatively narrow.

To summarize, our results indicate that the CdSe-PbSe heterostructures and PbSe NPLs exhibit strong confinement along the thickness direction, different from QDs with similar crystal sizes. Moreover, because of the very large exciton Bohr radius of PbSe (46 nm),^40,43^ relatively large redshifts in emission on the order of 100 meV are observed when the PbSe domains grow in size.

To further investigate the optical characteristics of the PbSe NPLs and CdSe-PbSe heterostructures, temperature-dependent photoluminescence spectra were measured. Emission spectra between 300 and 4 K are shown in Figure 7. Interestingly, the emission peak position for the 0.5 min cation-exchanged sample blueshifts ( < 0, Figure 7d) with decreasing temperature, while for the two samples with larger PbSe domains (5 and 420 min samples, Figure 7e,f), a redshift ( > 0) of the emission peak is observed when the temperature is decreased.^44^ For the smallest PbSe NCs (0.5 min), the lattice contraction at low temperatures increases quantum confinement, resulting in a blueshift of the band gap energy. Remarkably, this effect is sufficiently strong that it reverses the redshift observed for large PbSe NCs and bulk PbSe.^44^

If we compare the temperature dependence of our 2D (hetero)NPLs to earlier works on PbSe QDs,^45^ the 0.5 min cation-exchanged CdSe-PbSe heterostructures have a similar  value and emission peak position as 2 nm PbSe QDs (Figure S11a). The PbSe NPLs from the 5 and 420 min cation exchange have markedly higher  values than those reported for PbSe QDs with similar band gap energies (Figure S11b).^40,42^ Clearly, the anisotropic shape and quantum confinement along the thickness direction of the NPLs strongly influences . The present results for 2D PbSe nanostructures where confinement is much stronger in the thickness directions may help to better understand the contribution of lattice contraction on the exciton energy in PbSe NCs.

To obtain information on the exciton dynamics related to temperature-dependent radiative and nonradiative processes, time-resolved photoluminescence emission measurements were performed between 4 and 300 K. The luminescence decay curves measured for the three types of samples at different temperatures and at different emission wavelengths show a multiexponential behavior that can be accurately described by a two-exponential fit (Figure S12). The multiexponential behavior indicates that there are differences between NCs and complicates the analysis.

**Figure 8:**
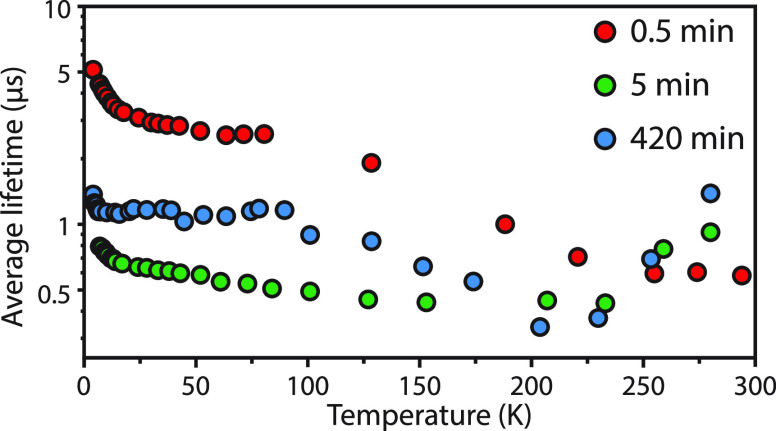
Temperature dependence of the average lifetimes for CdSe-PbSe heterostructures after 0.5, 5, and 420 min of cation exchange, determined from the decay curves (Figure S12). Note that the *y*-axis is given on a logarithmic scale. The samples were excited at 445 nm, while the emission was monitored at the emission peak maximum (see Figure 7a–c).

In order to compare the lifetimes of PbSe domains after 0.5, 5, and 420 min of cation exchange, we calculated average lifetimes using , and the results are shown in Figure 8. For the smallest PbSe NCs (0.5 min sample), the lifetime decreases from 75 to 300 K. This is different from the larger NCs (5 min sample) that show a decrease in lifetime between 75 and 200 K, followed by an increase in lifetime above 200 K. A similar increase in lifetime above 200 K for PbSe NPLs was reported before by Skurlov et al.^46^ Although it is beyond the scope of this study to explain the complex temperature-dependent lifetime behavior of these (hetero)NCs, the absence of clear trends between 75 and 300 K indicate the presence of thermally activated processes, including trapping and detrapping of charge carriers.^47^

Different from the lifetime behavior between 75 and 300 K, a clear lengthening of lifetime is observed for all three of the samples below 30 K. A sharp increase in lifetime occurs with a different temperature onset for the different PbSe structures. For the smallest PbSe domains (0.5 min sample), the lifetime increases from 3 to 5 μs by cooling the sample from 30 to 4 K. For the slightly larger domains and fully exchanged NPLs (5 and 420 min samples), the onsets are present at 22 and 10 K, respectively. The increase in lifetime is roughly linear in the log plot, consistent with a dynamic equilibrium between a dark and bright exciton state. A dark-bright splitting causes a lengthening of the radiative lifetime due to the forbidden character of the dark transition and is strongly size-dependent.^47−50^ In the PbSe NPLs, a larger dark-bright splitting is present than what is expected based on the lateral dimensions. Although the large contribution of nonradiative processes to the decay dynamics makes it difficult to accurately determine the dark-bright state splitting, it is nonetheless clear that the splitting is dominated by strong confinement along the thickness direction.

## Conclusion

To conclude, we have followed the Pb^2+^-for-Cd^2+^ cation exchange with PbBr_2_–OLAM on CdSe NPLs into 2D CdSe-PbSe heterostructures and PbSe NPLs by combining optical spectroscopy with HAADF-STEM imaging. Lowering the reaction temperature resulted in a slow-down of the cation exchange, yielding partially exchanged CdSe NPLs containing well-defined PbSe domains, with absorption and emission features from both CdSe and PbSe. Atomically resolved HAADF-STEM revealed the conservation of the selenium framework and the presence of well-defined {001} and {011} heterointerfaces between the zb-CdSe and rs-PbSe domains, similar to systems prepared with solid-state techniques. Performing the cation exchange procedure on CdSe quantum rings resulted in the formation of optically active PbSe quantum rings while the shape of the original NC was preserved, showing that this procedure is also applicable to other NC shapes.

Moreover, this work reports on the temperature-dependent optical properties of heterostructured CdSe-PbSe NPLs. Quantum confinement strongly influences the emission peak maximum of PbSe NPL domains, at longer growth times (>5 min of cation exchange) predominantly along the thickness direction. The smallest PbSe domains (0.5 min) show a negative , which is in agreement with similarly sized PbSe QDs. The increase of the  for larger PbSe NPLs (5 and 420 min of cation exchange) is stronger than for QDs with the same emission peak maximum, indicating a strong influence of the anisotropic shape. A clear increase of the lifetimes is observed upon cooling from 30 to 4 K for all PbSe nanostructures, indicative of dark-bright exciton splitting, which is strongly affected by the anisotropic confinement in the PbSe NPLs.

## Supporting Information Available

Calculation of the Pb^2+^-to-Cd^2+^ ratio during the reaction; photoluminescence absorption and emission spectra of samples prepared at 80, 60, 40, and 25 °C; BF-TEM imaging of a partially exchanged sample; elemental composition of CdSe-PbSe heterostructures, PbSe NPLs and PbSe quantum rings; temperature-dependent spectroscopy, including photoluminescence decay curves and a comparison to literature (PDF)
