# Supplementary material for: Two-Dimensional CdSe-PbSe Heterostructures and PbSe Nanoplatelets: Formation, Atomic Structure, and Optical Properties
Source: J Phys Chem C Nanomater Interfaces. 2022 Jan 17;126(3):1513–22. doi: 10.1021/acs.jpcc.1c09412 (PMC8802322; doi:10.1021/acs.jpcc.1c09412)
Supplement: Supplementary file 1 — jp1c09412_si_001.pdf [file jp1c09412_si_001.pdf]

# Two-Dimensional CdSe-PbSe Heterostructures and PbSe Nanoplatelets: Formation, Atomic Structure and Optical Properties – Supporting Information

*Bastiaan B.V. Salzmann,†‡ Jur de Wit,†‡ Chen Li,┴ Daniel Arenas-Esteban,┴ Sara Bals,┴ Andries Meijerink,† Daniel Vanmaekelbergh†**

†Condensed Matter & Interfaces, Debye Institute for Nanomaterials Science, Utrecht University, 3508TA Utrecht, The Netherlands. ┴EMAT and Nanolab Centre of Excellence, Antwerp University, 2020 Antwerp, Belgium. *E-mail: d.vanmaekelbergh@uu.nl. ‡These authors contributed equally.

**Section S1: Estimation of the $Pb^{2+}$ to $Cd^{2+}$ ratio**

To estimate the $Pb^{2+}$:$Cd^{2+}$ cation ratio at the beginning of the cation exchange reaction, we determine the amount of $Cd^{2+}$ cations in the CdSe NPLs from the shape of the nanocrystal and the absorption spectrum of the diluted stock dispersion by using Beer's law ($c = \frac{A_{300} \cdot d}{\varepsilon_{300} \cdot l}$); with $c$ the concentration of the CdSe NPLs (mol·L$^{-1}$), $A_{300}$ the absorbance of the nanocrystal dispersion at 300 nm ($A_{300}=2.722$), $d$ the dilution factor of the dispersion ($d=101$), $\varepsilon_{300}$ the absorption coefficient of the nanocrystals at 300 nm (in $cm^{-1} \cdot mol^{-1} \cdot L$), and $l$ the path length of the cuvette (1 cm). In here, the absorption coefficient ($\varepsilon_{300}$) is related to the absorption cross section of a single nanoplatelet ($\sigma_{300}$) at 300 nm as $\varepsilon_{300} = \sigma_{300} \frac{N_{av}}{\ln(10)}$, with $N_{av}$ Avogadro's constant.[1] We calculated the absorption cross section $\sigma_{300}$ using a previously reported protocol from Geiregat et al.[2] where the bulk dielectric values of CdSe and shape of the nanocrystal (see below) were taken into account. This results in respectively $\sigma_{300} = 1.28 \cdot 10^{-13}\ cm^{-2}$ and $\varepsilon_{300} = 3.34 \cdot 10^{7}\ cm^{-1} \cdot M^{-1}$, in line with theoretical and experimental literature values,[2, 3] yielding a concentration $c = \frac{2.722 \cdot 101}{3.34 \cdot 10^{7} \cdot 1} = 8.22 \cdot 10^{-6}$ *mol CdSe NPLs*$\cdot L^{-1}$. We added 210 µL of this concentrated stock solution to each experiment, which corresponds to $1.73 \cdot 10^{-9}$ *mol CdSe NPLs*.

We "fill" the shape of a single CdSe NPL with unit cells of zinc blende CdSe to estimate the number of $Cd^{2+}$ cations per nanocrystal. The lateral sizes determined from HAADF-STEM images are 11.9 by 14.7 nm$^2$, see also Figure 2a of the main text. As the NPLs exhibit the characteristic (HH,e) and (LH,e) absorption features of 4.5 ML thick CdSe NPLs at 2.42 and 2.58 eV (Figure 2g), the nanocrystals consist of four monolayers of CdSe and a terminating

layer of Cd along the thickness direction.[4, 5] Figure S1 depicts a schematic representation of this crystal structure along the thickness direction, showing that this matches with a building block of two stacked unit cells of zinc blende CdSe which contain 10 $Cd^{2+}$ cations and 8 $Se^{2-}$ anions. As the lattice constant of zinc blende is 0.6077 nm, the nanocrystals consist of on average 20 by 24 “building blocks” of two stacked unit cells of CdSe, resulting in respectively 4800 $Cd^{2+}$ and 3840 $Se^{2-}$ ions per CdSe NPL. This corresponds to the presence of $1.73 \cdot 10^{-9}$ *mol CdSe NPLs* $\cdot$ *4800* $Cd^{2+}$ *cations/NPL* = $8.29 \cdot 10^{-6}$ *mol* $Cd^{2+}$ *ions*.

As we add $65 \cdot 10^{-6}$ *mol* $Pb^{2+}$ (from 24 mg $PbBr_2$) and $8.29 \cdot 10^{-6}$ *mol* $Cd^{2+}$ (from the CdSe NPLs) to the reaction mixture, an approximate $Pb^{2+}$:$Cd^{2+}$ ratio of 7.8:1 is present during the cation exchange reaction. Although several assumptions have been made for this calculation, we can safely conclude that an excess amount of $Pb^{2+}$ ions is present to drive the $Pb^{2+}$-for-$Cd^{2+}$ cation exchange in the CdSe NPLs.

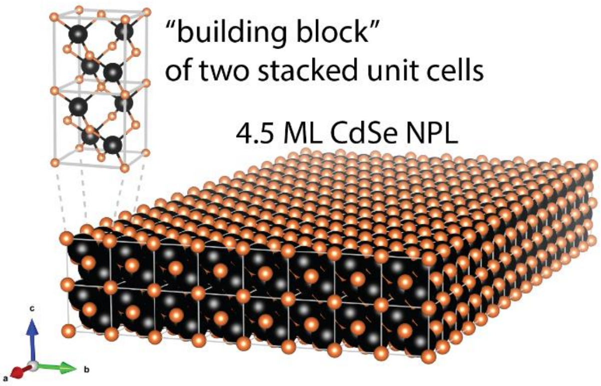


**Figure S1.** Schematic representation of a 4.5 ML thick CdSe NPL, showing that the crystal structure along the thickness direction has “building blocks” of two stacked unit cells of zinc blende CdSe. A single building block contains 10 $Cd^{2+}$ and 8 $Se^{2-}$ ions.

## Section S2: Optical spectroscopy and structural characterization

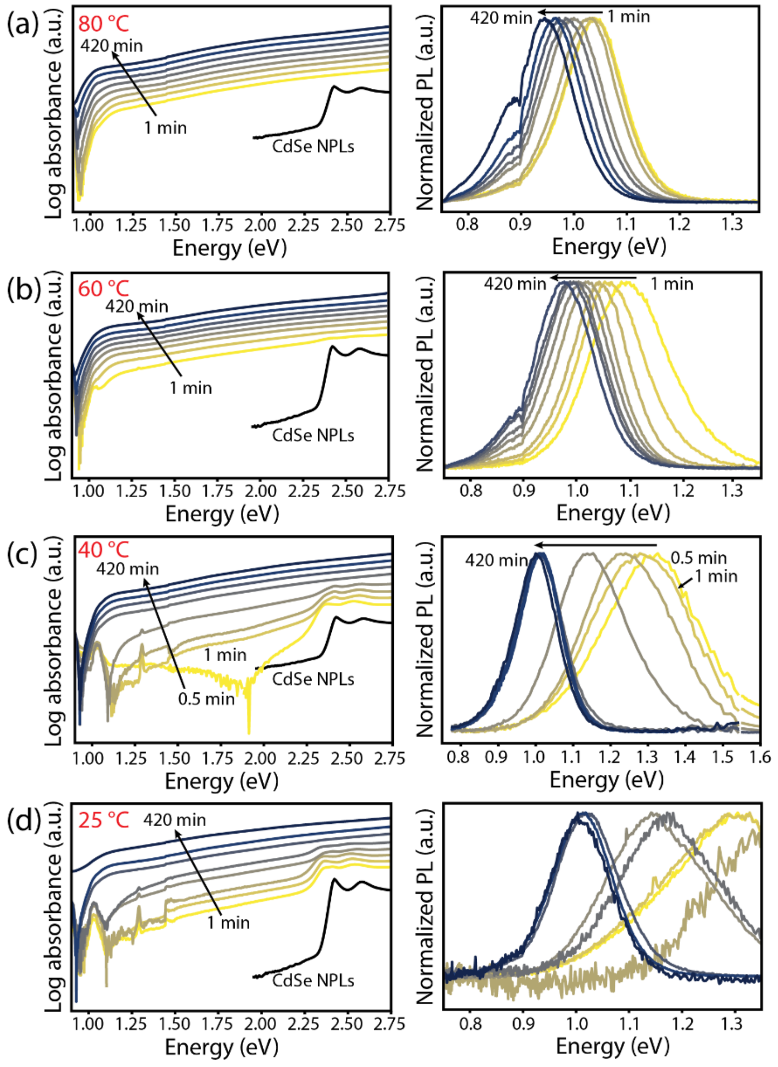


**Figure S2.** Absorption and emission spectra of aliquots taken during the $Pb^{2+}$-for-$Cd^{2+}$ cation exchange of CdSe NPLs into PbSe NPLs at 80, 60, 40 and 25 °C for up to 420 min of reaction. The absorption data is plotted on a logarithmic scale to visualize the absorption of PbSe.

The absorption spectrum of the aliquot taken after 1 min of reaction time at 80 °C (Figure S2a) shows the absence of characteristic features of the (HH,e) and (LH,e) transitions from CdSe NPLs at 2.42 and 2.58 eV. However, an onset at 1.1 eV is present, that shifts during the 420 min of reaction to 1.0 eV. Except for this onset, no other features of PbSe are visible, in agreement to previous results on PbX (X=S, Se) NPLs.[6, 7] The emission spectra show a comparable redshift as the absorption spectra: the emission peak after 1 min of reaction at 1.05 eV has been shifted to 0.95 eV after 420 min. Analogous trends in absorption and emission are observed at 60 °C, although the shifts to lower energies are slightly smaller (Figure S2). Decreasing the temperature to 40 °C shows similar trends in absorption and emission as 60 and 80 °C, albeit the (HH,e) and (LH,e) absorption transitions from 4.5 ML thick CdSe NPLs are visible at the early stages of the reaction. Simultaneously, an increasing broad absorption band appears between 1.25 and 2.25 eV due to the growth of PbSe domains. A lower temperature of 25 °C did result in irreproducible results. The glovebox heated up during the cation exchange reaction which consequently reduced the ability to control the reaction temperature. Moreover,

the recorded emission spectra showed low intensities, suggesting that a temperature of 40 °C or higher anneals nanocrystals structure *in situ*, thereby reducing the number of defects in the crystal lattice.

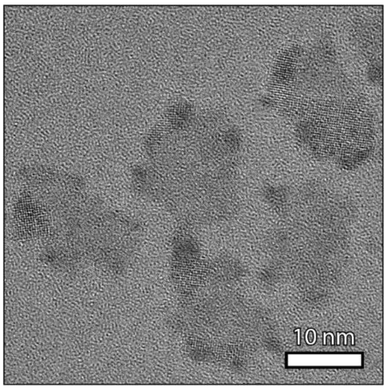


**Figure S3.** High resolution BF-TEM image of an aliquot taken after 5 min of cation exchange at 40 °C. Domains of rock salt PbSe are clearly visible at the edges of the nanocrystals and are occasionally in zone axis. In contrast to the HAADF-STEM image (Figure 2d), the difference between the low-contrast CdSe and background is not as clear due to the additional noise from the formvar layer of the EM grid.

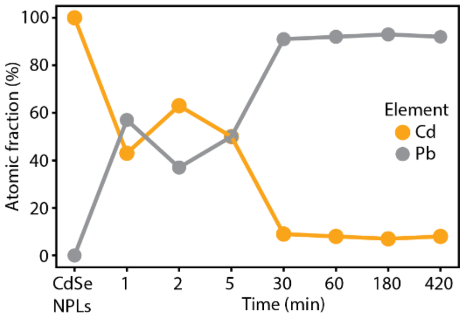


**Figure S4.** Elemental composition of the heterostructures during the conversion of CdSe NPLs into PbSe NPLs determined with EDX in BF-TEM mode. We remark that elemental analysis with EDX is relatively inaccurate as multiple factors influence the measurements.

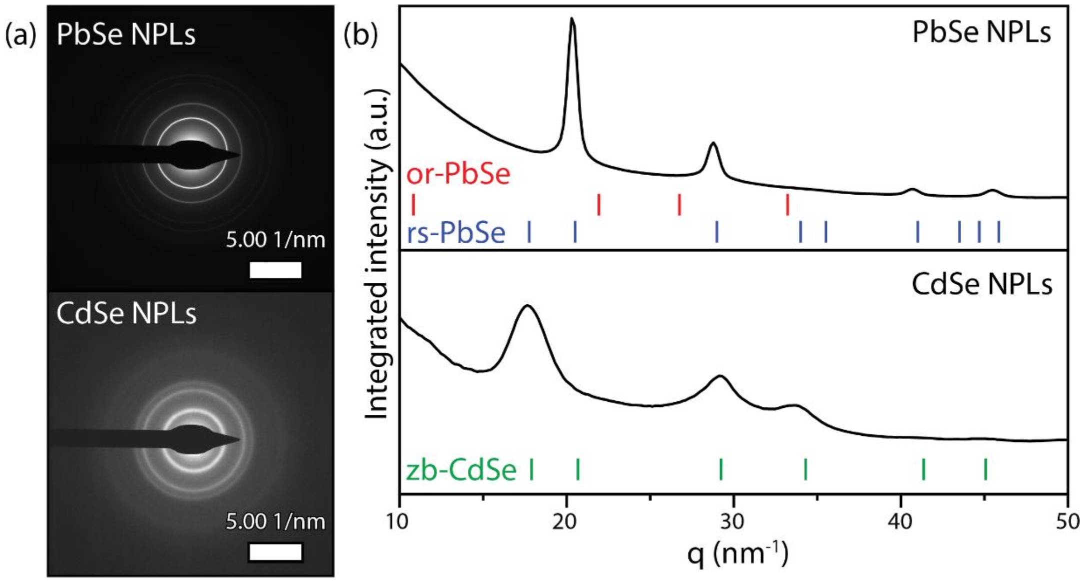


**Figure S5.** SAED diffraction patterns (a) and rotationally averaged patterns (b) of CdSe and PbSe NPLs, together with reference patterns of zb-CdSe, rs-PbSe and orthorhombic PbSe (or-PbSe). The SAED pattern of the starting CdSe NPLs is in agreement with zb-CdSe whereas the pattern of the PbSe NPLs matches that of rs-PbSe.

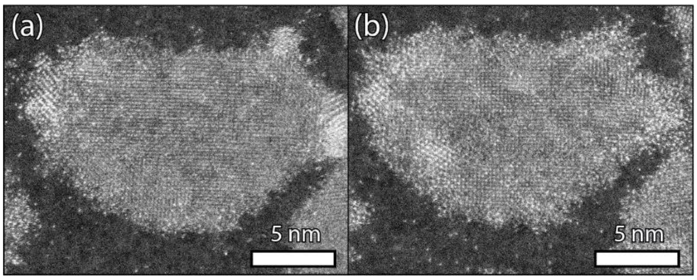


**Figure S6.** High resolution HAADF-STEM images of a partially exchanged nanocrystal (0.5 min aliquot at 40 °C) after the first (a) and second scan (b), showing that the crystal structure has been deformed under influence of the electron beam. In order to minimize these structural changes during imaging, low beam currents (~ 5 pA) and magnifications were used.

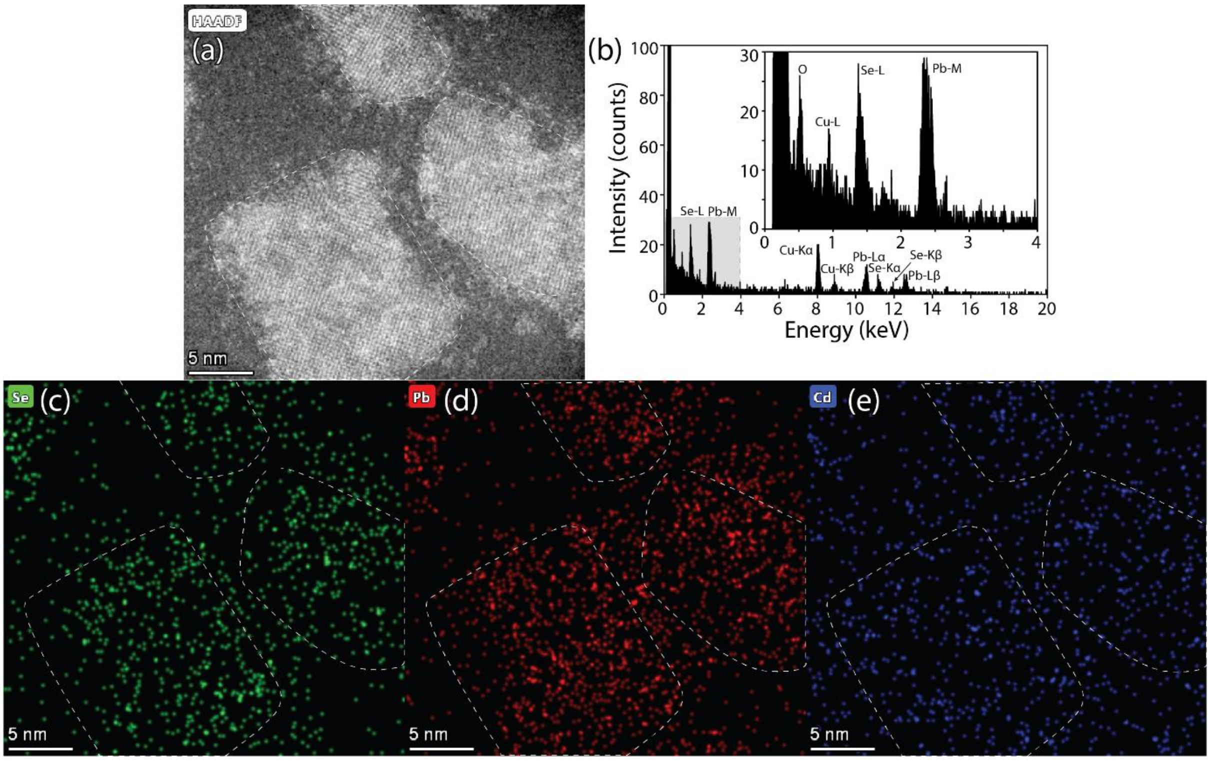


**Figure S7.** EDX in high resolution HAADF-STEM mode on several PbSe NPLs after $Pb^{2+}$-for-$Cd^{2+}$ exchange for 420 min at 40 °C. Although a homogenous distribution of blue dots of cadmium is visible (e), no peak of Cd is present in the EDX spectrum (at 3.1 keV). Therefore, the detected X-rays are originated from background noise from the EDX detector and no detectable amount of Cd is present.

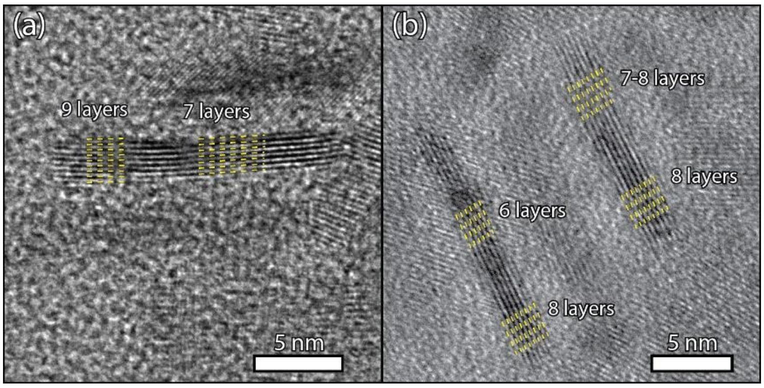


**Figure S8.** BF-TEM imaging of PbSe NPLs with an edge-up configuration, prepared by cation-exchange for 420 min at 40 °C. Although the nanocrystals are not perfectly in zone axis, the dashed yellow lines indicate the layered structure of rs-PbSe which varies between 6 and 9 layers of PbS.

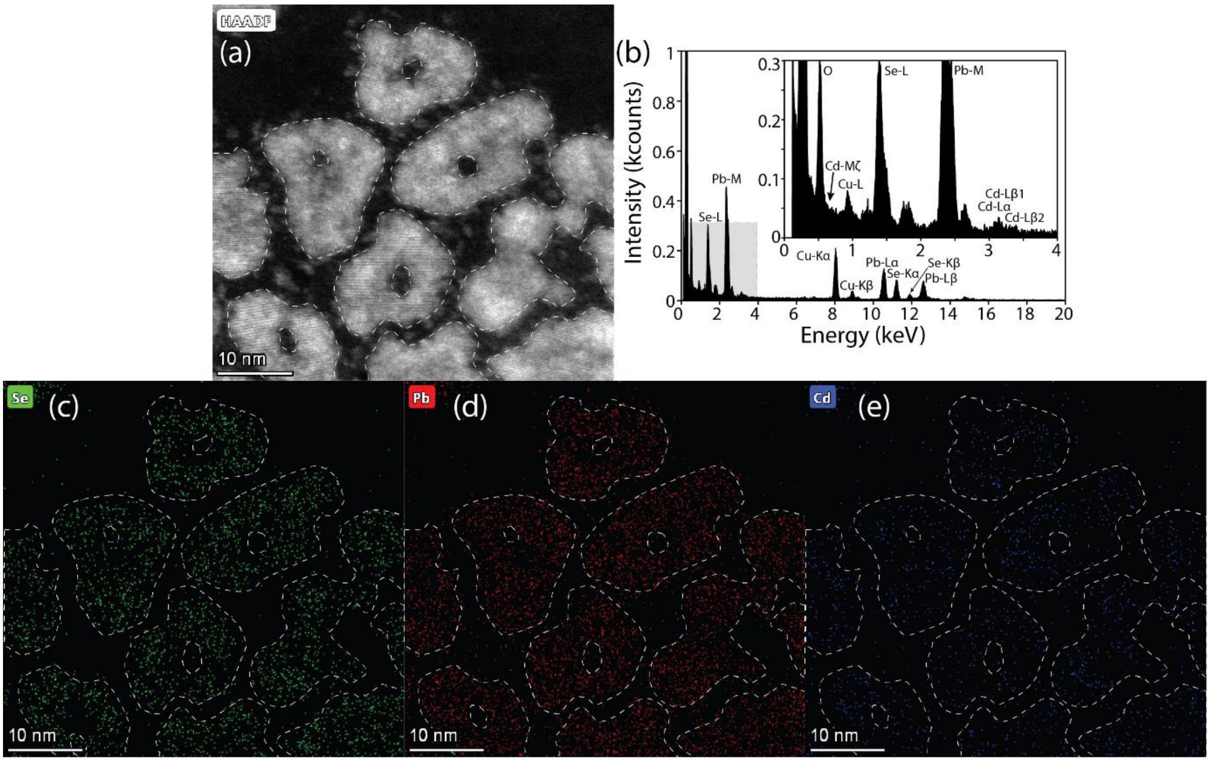


**Figure S9.** EDX in high resolution HAADF-STEM mode on PbSe quantum rings after performing $Pb^{2+}$-for-$Cd^{2+}$ cation exchange for 420 min on CdSe quantum rings. From the EDX spectrum, atomic ratios of 45:4:51 for Pb:Cd:Se were found.

**Section S3: Temperature-dependent spectroscopy**

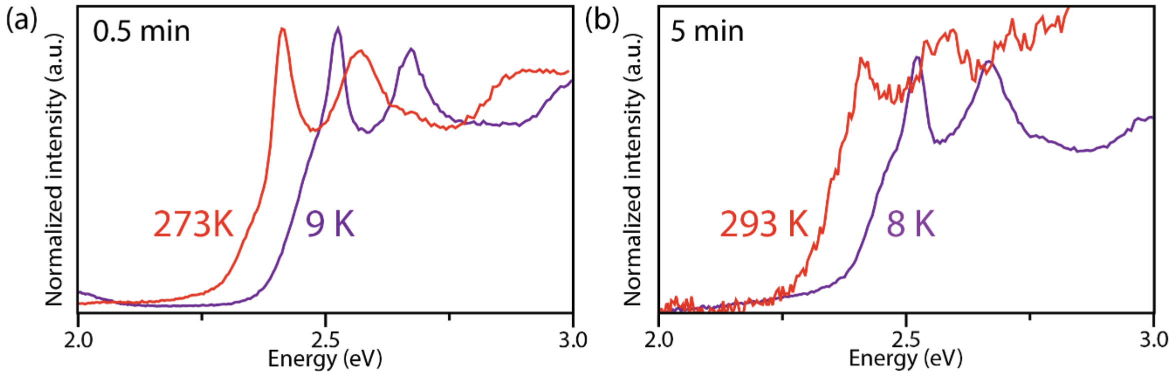


**Figure S10.** Excitation spectra while monitoring the PbSe emission for 0.5 (a) and 5 min (b) samples. The emission was recorded at the high energy side of the PbSe emission band, respectively at 1.85 and 1.50 eV. Both after 0.5 and 5 min exchange the room temperature, the excitation spectra reveal the characteristic (HH,e) and (LH,e) transitions of 4.5 ML thick CdSe NPLs, indicating the presence of 4.5 ML thick CdSe domains and efficient energy transfer from CdSe to PbSe. Below 2.45 eV there is a weak absorption onset visible typical for above bandgap PbSe absorption. At cryogenic temperatures the excitation spectrum has been blue-shifted by ~0.12 eV, in agreement with previous results for 4.5 ML thick CdSe NPLs.[8]

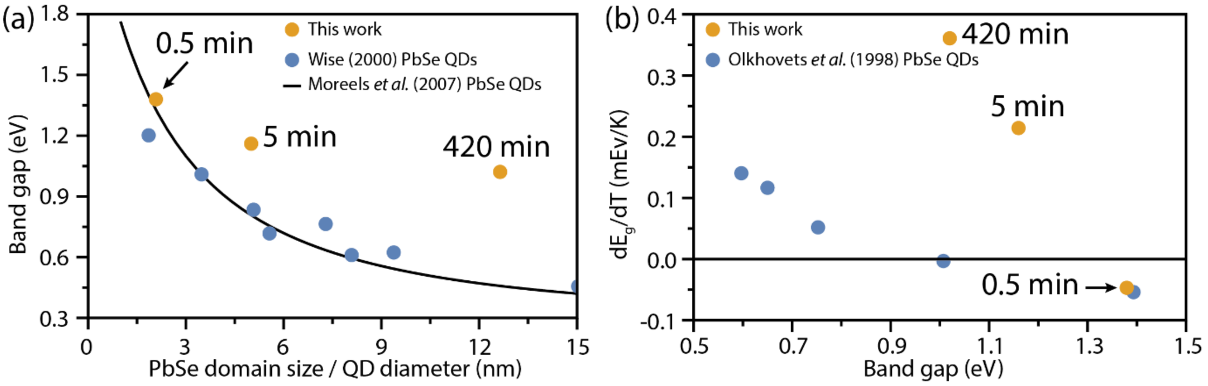


**Figure S11.** Comparison of the band gap at 300 K and change of band gap as a function of temperature ($\frac{dE_g}{dT}$) of our 2D CdSe-PbSe heterostructures (0.5 and 5 min samples) and PbSe NPLs (420 min sample) to literature values.[9, 10] (a) The band gap energy of PbSe QDs and two-dimensional PbSe-CdSe heterostructures and PbSe NPLs nanostructures after cation exchange. The band gap energy of the smallest PbSe NCs/domains (0.5 min sample) is in good agreement with previous results for PbSe QDS. Larger PbSe NPLs (5 and 420 min sample) experience stronger confinement in the thickness direction and therefore have a higher band gap energy than one would expect for PbSe QDs with a similar size. (b) Sizing curve from Moreels *et al.*[10] is used to convert PbSe QD diameter versus $\frac{dE_g}{dT}$ values from Olkhovets *et al.* into $E_g$ versus $\frac{dE_g}{dT}$ values.[11] Again, the smallest PbSe domain values are in good agreement with previous results, while the laterally larger PbSe NPLs have higher $\frac{dE_g}{dT}$ values. The difference is insightful, as the lattice contraction effect on the exciton energy as a function of temperature of PbSe NPLs clearly different from PbSe QDs.

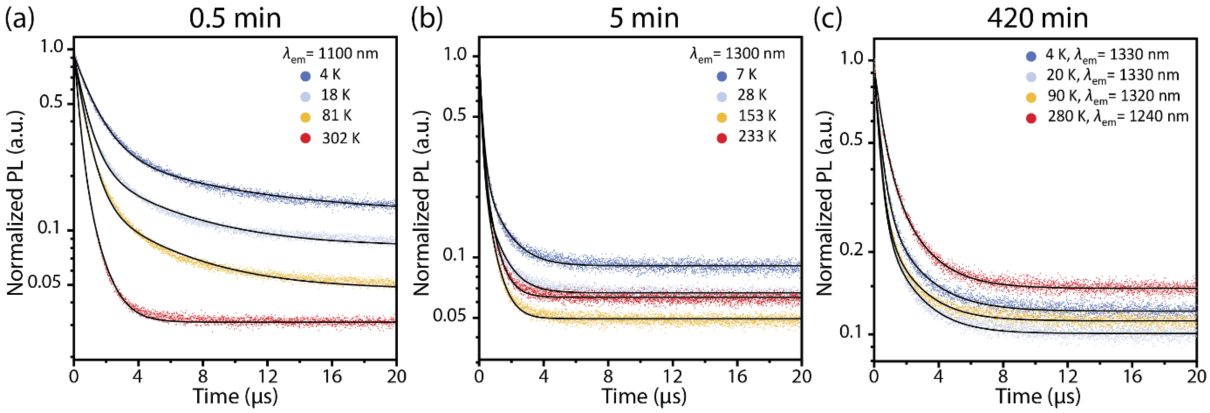


**Figure S12.** Photoluminescence decay curves of CdSe-PbSe heterostructures and PbSe NPLs after $Pb^{2+}$-for-$Cd^{2+}$ for (a) 0.5, (b) 5 and (c) 420 min of cation exchange for pulsed 445 nm excitation (pulse width: 180, 120 and 120 ns). All decay curves are clearly multi-exponential as a result of the large polydispersity and reflect variations in radiative and non-radiative decay rates within samples. The black lines are two-exponential fits in the form $I\ (t) = A1 * e^{-t/\tau1} + A2 * e^{-t/\tau2}$, from which the average exciton lifetimes are calculated using the relation $\tau_{av} = \frac{A_1{\tau_1}^2+A_1{\tau_2}^2}{A_1\tau_1+A_2\tau_2}$(shown in Figure 8).

## References


1. Hens, Z.; Moreels, I., Light absorption by colloidal semiconductor quantum dots. *Journal of Materials Chemistry* **2012,** 22, (21).
2. Geiregat, P.; Tomar, R.; Chen, K.; Singh, S.; Hodgkiss, J. M.; Hens, Z., Thermodynamic Equilibrium between Excitons and Excitonic Molecules Dictates Optical Gain in Colloidal CdSe Quantum Wells. *J Phys Chem Lett* **2019,** 10, (13), 3637-3644.
3. Yeltik, A.; Delikanli, S.; Olutas, M.; Kelestemur, Y.; Guzelturk, B.; Demir, H. V., Experimental Determination of the Absorption Cross-Section and Molar Extinction Coefficient of Colloidal CdSe Nanoplatelets. *The Journal of Physical Chemistry C* **2015,** 119, (47), 26768-26775.
4. Achtstein, A. W.; Antanovich, A.; Prudnikau, A.; Scott, R.; Woggon, U.; Artemyev, M., Linear Absorption in CdSe Nanoplates: Thickness and Lateral Size Dependency of the Intrinsic Absorption. *The Journal of Physical Chemistry C* **2015,** 119, (34), 20156-20161.
5. Ithurria, S.; Tessier, M. D.; Mahler, B.; Lobo, R. P.; Dubertret, B.; Efros, A. L., Colloidal nanoplatelets with two-dimensional electronic structure. *Nat Mater* **2011,** 10, (12), 936-41.
6. Akkerman, Q. A.; Martín-García, B.; Buha, J.; Almeida, G.; Toso, S.; Marras, S.; Bonaccorso, F.; Petralanda, U.; Infante, I.; Manna, L., Ultrathin Orthorhombic PbS Nanosheets. *Chemistry of Materials* **2019,** 31, (19), 8145-8153.
7. Manteiga Vazquez, F.; Yu, Q.; Klepzig, L. F.; Siebbeles, L. D. A.; Crisp, R. W.; Lauth, J., Probing Excitons in Ultrathin PbS Nanoplatelets with Enhanced Near-Infrared Emission. *J Phys Chem Lett* **2021**, 680-685.
8. van der Bok, J. C.; Dekker, D. M.; Peerlings, M. L. J.; Salzmann, B. B. V.; Meijerink, A., Luminescence Line Broadening of CdSe Nanoplatelets and Quantum Dots for Application in w-LEDs. *The Journal of Physical Chemistry C* **2020,** 124, (22), 12153-12160.
9. Wise, F. W., Lead salt quantum dots: the limit of strong quantum confinement. *Acc Chem Res* **2000,** 33, (11), 773-80.
10. Moreels, I.; Lambert, K.; De Muynck, D.; Vanhaecke, F.; Poelman, D.; Martins, J. C.; Allan, G.; Hens, Z., Composition and size-dependent excition coefficient of colloidal PbSe QDs. *Chemistry of Materials* **2007,** 19, (25), 6101-6106.
11. Olkhovets, A.; Hsu, R. C.; Lipovskii, A.; Wise, F. W., Size-Dependent Temperature Variation of the Energy Gap in Lead-Salt Quantum Dots. *Physical Review Letters* **1998,** 81, (16), 3539-3542.
